# Commentary: Research status and prospects of acupuncture for autism spectrum disorders

**DOI:** 10.3389/fpsyt.2023.1179048

**Published:** 2023-05-25

**Authors:** Antonio M. Persico

**Affiliations:** Child and Adolescent Neuropsychiatry Program, Modena University Hospital & Department of Biomedical, Metabolic and Neural Sciences, University of Modena and Reggio Emilia, Modena, Italy

**Keywords:** acupunture, autism spectrum disorder, complementary and alternative approaches, evidence-based therapies, non-conventional therapies, experimental methodology and design, editorial policies

## Introduction

In this Issue, readers of the Frontiers in Psychiatry—Autism section will find the article “*Research Status and Prospects of Acupuncture for Autism Spectrum Disorders*” by Li et al. (1). In this article the Authors provide a broad and thorough overview of the current status of research addressing the efficacy and safety of acupunture in Autism Spectrum Disorder (ASD). This manuscript has sparked debate in our Editorial Board as to whether our journal should publish articles regarding therapeutic approaches which are not evidence-based at this time. Indeed, evidence supporting the efficacy of acupunture in ASD is still scanty and studies are in most instances flawed by methodological limitations which raise caution in interpreting their results. An additional layer of complexity is added by the lack of knowledge about the theoretical background and the practical management of acupunture outside Chinese culture, as this approach is usually not part of medical training and academic teaching elsewhere in world.

## Acupuncture as an alternative therapeutic approach

Acupunture is just one among many therapeutic approaches not supported by sufficient scientific evidence of efficacy and safety in ASD. Yet, these therapies are not forbidden and they are already available to autistic individuals and their families on a private basis. On the one hand, non evidence-based therapies cannot be recognized by National Health Systems, which in the vast majority of countries understandably neither provide them, nor cover their costs. On the other hand, parents cannot be blamed for searching for an effective cure if their autistic child does not show sufficient behavioral improvement applying currently available behavioral and medical therapies. Families are entitled to foster hope and to seek relief. Scientific research definitively supporting their validity or disconfirming their efficacy is the only serious mean to combat an unacceptable market of “magic therapeutic bullets”, which benefits only speculators while imposing an additional economic burden on families.

## General guidelines for articles on alternative therapeutic approaches

For all these reasons, the need to stimulate replicable and valid scientific research on the efficacy and safety of therapies for ASD, including evidence-based and non-conventional interventions, should be recognized. At the same time, Frontiers in Psychiatry–Autism Section shall apply the same high quality threshold for the acceptance of manuscripts regarding non-conventional therapies, as for evidence-based therapies. This is a brief list of general guidelines for investigators possibly interested in submitting their work on non-conventional therapies to Frontiers in Psychiatry–Autism Section in the future:

- Clinical trials are welcome, but special attention will be placed on their methodology: accurate design, appropriate control and randomization, sample size determination by power analysis based on reliable estimates of effect size and variance, sensitive and reliable primary and secondary outcome measures, strategies to minimize the placebo effect, safety measures, appropriate trial duration and sufficient time points for clinical assessment will all be sought in randomized control trials of therapeutic interventions (2–4). Much-needed objective biomarkers able to identify responders to a given treatment will also represent a major asset, though not yet mandatory at this time (5).
- Pre-clinical studies addressing non-conventional therapies, whether employing cellular or animal models, or mechanistic human studies, must be based on a solid theoretical background and employ methodological constructs able to provide reliable evidence endowed with sufficient validity in humans (6, 7). Only this high-quality preclinical work will later foster clinical trials.
- A limited number of systematic reviews focused on some of the major non-conventional autism therapies may also be of interest. In addition to summarizing all available Literature, these reviews will have to clearly state that “all available data is not sufficient to support the clinical effectiveness of this non-conventional therapy in ASD and to justify its use in clinical practice”. A detailed description of the methodological limitations of published studies will be especially useful.

## Discussion

There is a broad consensus both in academia and within national health systems that therapeutic interventions in ASD should be based on empirical evidence of efficacy and safety. Indeed, empirically supported treatments (ESTs) must have been proven effective and safe in well-designed randomized controlled trials (RCTs) and/or in single-subject experiments, by comparison with a sham or active treatment condition (8, 9). Once a critical mass of well-designed empirical studies has been published in peer-reviewed journals, the second step typically consists in collecting and harmonizing this information into systematic reviews that define ESTs and their relative evidence level, i.e., “efficacious and specific”, “efficacious”, or “possibly efficacious” (8, 9). This, in turn, allows knowledge to be ultimately transferred “from bench to bedside” through appropriate education and training programs designed for medical and health personnel. Each of these three steps builds upon the previous one; however, solid and rigorous individual studies providing replicable and valid evidence clearly represent the foundation of the entire process. The definition of Evidence-Based Practices (EBPs) concerning behavioral interventions endowed with efficacy in children and adolescents with ASD has reached the second step already some years ago (10), and the dynamic flow of novel empirical evidence has already required updating data and refining conclusions (11). Pharmacological interventions are perhaps lagging a little behind behavioral interventions, but have also recently reached the stage whereby systematic reviews can indeed provide a comprehensive overview of a sufficient critical mass of RCTs performed in the pediatric population (12). Both for behavioral and pharmacological interventions, in recent years this knowledge has begun transferring into the clinical setting and is slowly but steadily improving the clinical management of children and adolescents with ASD.

Non-conventional therapies, spanning from supplements and vitamins to probiotics, heavy metal chelators, diets, massage, hyperbaric oxygen, stem cells, acupunture and others, have not yet fulfilled the first step of this process: the empirical evidence supporting their efficacy is either lacking, insufficient, or of low quality. Therefore, also systematic reviews must be viewed as “work-in-progress” and not as “conclusive evidence”. Li et al. (1) have revised their manuscript according to these principles, and we have gladly accepted it for publication, as it presents state-of-the-art information on a non-conventional approach of interest to many patients with ASD and their families, both in China and throughout the world. It can only be detrimental that science self-imposes limitations on its scope based on prejudice: any plausible therapeutic approach, provided it is safe for the patients, may yield some benefit and should be the object of well-designed studies, until proven uneffective. This position does NOT make Frontiers in Psychiatry - Autism Section a supporter of the use of acupunture in ASD, which we indeed discourage until conclusively proven effective, nor an ideal target journal for manuscripts on non-conventional therapies. While we do remain open to these type of contributions, we shall indeed apply a careful policy, accurately scrutinizing submissions along the lines summarized above. At the same time, we believe that only attracting “non-conventional therapies” into the realm of science, will they one day become “evidence-based”, if effective, or be definitively abandoned, if proven uneffective or unsafe.

## Author contributions

The author confirms being the sole contributor of this work and has approved it for publication.
